# Genomics of Sorghum

**DOI:** 10.1155/2008/362451

**Published:** 2008-04-23

**Authors:** Andrew H. Paterson

**Affiliations:** Plant Genome Mapping Laboratory, Department of Crop and Soil Sciences, University of Georgia, 111 Riverbend Road, Athens, GA 30602, USA

## Abstract

Sorghum (*Sorghum bicolor* (L.) Moench) is a subject of plant genomics research based on its importance as one of the world's leading cereal crops, a biofuels crop of high and growing importance, a progenitor of one of the world's most noxious weeds, and a botanical model for many tropical grasses with complex genomes. A rich history of genome analysis, culminating in the recent complete sequencing of the genome of a leading inbred, provides a foundation for invigorating progress toward relating sorghum genes to their functions. Further characterization of the genomes other than Saccharinae cereals may shed light on mechanisms, levels, and patterns of evolution of genome size and structure, laying the foundation for further study of sugarcane and other economically important members of the group.

## 1. WHY SORGHUM?

As a food and feed crop, sorghum is an important “failsafe” in the global agroecosystem. Worldwide, sorghum is the 5th most important grain crop grown based on tonnage, after maize, wheat, rice, and barley (www.fao.org). Sorghumis unusually tolerant of low input levels, an essential trait for areas such as Northeast Africa and the US Southern Plains that receive too little rainfall for most other grains. Increased demand for limited fresh water supplies, increasing use of marginal farmland, and global climatic trends, all suggest that dryland crops such as sorghum will be of growing importance to feed the world’s expanding populations.

Currently the 2nd source of grain-based ethanol in the US (after maize), sorghum is a biofuel crop of growing importance. The generally lower water demands and market price for sorghum than maize, versus their equal per-bushel ethanol yields, suggests that sorghum will be of growing importance in meeting grain-based biofuels needs. Cellulosic biofuel production offers compelling advantages over seed-based production [1], but will require greater utilization of marginal lands to make the low per-unit value of biomass production economical, and will be heavily dependent upon the use of perennials to be sustainable [2, 3]. A relatively advanced state of knowledge of the genetic control of perenniality in sorghum [4, 5] and early progress in functional genomics of perenniality [6] add to its promise as a cellulosic biofuels crop. “Sweet sorghums” with high sugar content in stems, already grown for forage and silage, may be especially promising.

The *Sorghum* genus also offers the opportunity to gain new insights into biology of weeds and invasives. Vegetative dispersal by rhizomes (underground stems) and seed dispersal by disarticulation of the mature inflorescence (shattering) cause “Johnsongrass” [*Sorghum halepense* (L.) Pers, 2*n* = 2*x* = 40] to rank among the world's most noxious weeds [7]. Johnsongrass is an interspecific hybrid of *Sorghum bicolor* and *S. propinquum*, the latter contributing rhizomatousness. *Sorghum bicolor* and *S. propinquum* are readily crossed, and their progeny provide a system in which to dissect the genetic basis of rhizomatousness [4]. The same features that make Johnsongrass such a troublesome weed are actually desirable in many forage, turf, and biomass crops which are genetically complex. Therefore, sorghum offers novel learning opportunities relevant to weed biology as well as to improvement of a wide range of other forage, turf, and biomass crops.

The small genome of sorghum has long been an attractive model for advancing understanding of the structure, function, and evolution of cereal genomes. Sorghum is representative of tropical grasses in that it has “C4” photosynthesis, using complex biochemical and morphological specializations to improve carbon assimilation at high temperatures. By contrast, rice is more representative of temperate grasses, using “C3” photosynthesis. Its lower level of gene duplication than many other tropical cereals makes sorghum, like rice, an attractive model for functional genomics. However, sorghum is much more closely related than rice to many major cereal crops with complex genomes and high levels of gene duplication. *Sorghum* and *Zea* (maize, the leading US crop with a farm-gate value of $15–20 billion/y) diverged from a common ancestor ~12 mya [8, 9] versus ~42 mya for rice and the maize/sorghum lineage [10]. *Saccharum* (sugarcane), arguably the most important biofuels crop worldwide, valued at ~$30 billion including $1 billion/y in the US*, may have shared ancestry with sorghum as little as 5 million years ago [11], retains similar gene order [12], and even produces viable progeny in some intergeneric crosses [13]. *Zea* has undergone one whole-genome duplication since its divergence from *Sorghum* [14], and *Saccharum* has undergone at least two [12].

## 2. PROGRESS IN SORGHUM GENOME CHARACTERIZATION

### 2.1. Genetic mapping

Linkage mapping in sorghum takes advantage of its straightforward diploid genetics, amenability to inbreeding, high levels of DNA polymorphism between *Sorghum* species, and manageable levels of DNA polymorphism within *S. bicolor*. High-density reference maps of one intraspecific *S. bicolor* [15–18] and one interspecific *S. bicolor* x *S. propinquum* [19, 20] cross provide about 2600 sequence-tagged-sites (based on low-copy probes that have been sequenced), 2454 AFLP, and ~1375 sequence-scanned (based on sequences of genetically anchored BAC clones) loci. These two maps share one common parent (*S. bicolor* “BTx623”) and are essentially colinear [21]. Cytological characterization of the individual sorghum chromosomes has provided a generally adopted numbering system [22].

More than 800 markers mapped in sorghum are derived from other taxa (hence serve as comparative anchors) and additional sorghum markers have been mapped directly in other taxa, or can be plotted based on sequence similarity. Anchoring of the sorghum maps to those of rice [10, 23], maize [20, 24], sugarcane [12, 25], millet [26], switchgrass [27], bermuda grass [28], and others provides for the cross-utilization of results to simultaneously advance knowledge of many important crops.

### 2.2. Physical mapping

Sorghum was the first angiosperm for which a BAC library was published [29]. Estimates of the physical size of the sorghum genome range from 700 Mbp based on Cot analysis [30] to 772 Mbp based on flow cytometry [31]. This makes the sorghum genome about 60% larger than that of rice, but only about 1/4 the size of the genomes of maize or human. DNA renaturation kinetic analysis [30] shows the sorghum genome to be comprised of about 16% foldback DNA, 15% highly repetitive DNA (with individual families occurring at an average of 5200 copies per genome), 41% middle-repetitive DNA (average 72 copies) and 24% low-copy DNA. About 4% of the DNA remained single-stranded at very high Cot values and is assumed to have been damaged (thus the other percentages are slight underestimates).

High-coverage BAC libraries are available for BTx623 (about 12X coverage from H*ind*III and 8X from B*am*HI), *S. propinquum* (13-14X coverage from E*co*RI (~7X) and H*ind*III (~7X) and IS3620C (~9X coverage from H*ind*III). A total of 69 545 agarose-based fingerprints from BTx623 BACs are also anchored with 211,558 hybridization loci from 7292 probes (about 2000 of which are genetically mapped). In parallel, 40 957 agarose-based fingerprints from *S. propinquum* are anchored with 189 735 hybridization loci from 7481 probes (2000 genetically mapped). Targeted HICF of additional contig-terminal BACs has been used to fill gaps. Each of these has been assembled into WebFPC-accessible physical maps (http://www.stardaddy.uga.edu/fpc/WebAGCoL/bicolor/WebFPC and http://www.stardaddy.uga.edu/fpc/WebAGCoL/propinquum/WebFPC), for which earlier versions have been described in detail [32]. About 456 *S. propinquum* and 303 *S. bicolor* BAC contigs (41% of BACs, 80% of single-copy loci) appear to be well-anchored to euchromatic regions, with the percentage of the genome attributable to euchromatin likely to rise with additional anchoring. The finding that 41% of BACs are anchored to euchromatin while only 24% of the sorghum genomic DNA is single- or low-copy [with an overall kinetic complexity of 1.64×10^8^ [30]], suggests that sorghum euchromatin includes a mixture of low-copy and repetitive DNA.

### 2.3. Genome sequence

The shotgun sequencing of a leading US sorghum inbred, BTx623, is now complete, with ~10.5 million reads (~8X coverage) deposited in the NCBI Trace Archive. Early analysis confirms that the sorghum genome sequence will be a suitable substrate for a complete and high-quality annotation. In a preliminary assembly (that is expected to further improve with ongoing analysis), more than 97% of sorghum protein-coding genes (ESTs) were captured in the ~250 longest scaffolds. The vast majority of these can be linked, ordered, and oriented using the genetic and physical map to reconstruct complete chromosomes. Alignments of the preliminary assembly to sorghum methyl-filtered sequence; sorghum, maize, and sugarcane transcript assemblies; and the *Arabidopsis* and rice proteomes confirms the base-level accuracy of the assembly and correct local structure of protein-coding loci.

Additional resources from reduced-representation sequencing will contribute to the identification of expressed portions of the genome sequence. The sorghum gene space is presently represented by approximately 204 000 expressed sequence tags, many of which have been clustered into ~22 000 unigenes representing more than 20 diverse libraries from several genotypes [33]. About 500 000 methyl-filtered (MF) reads that provide an estimated 1x coverage of the MF-estimated gene space [34] have been assembled into contigs (SAMIs, http://magi.plantgenomics.iastate.edu).

## 3. POSTGENOMICS OF SORGHUM

With the genome sequence available, one can anticipate renewed interest and accelerated progress in relating sorghum genes to their functions. Prior efforts will benefit from the sequence as a means of integrating diverse data types, providing for the formulation and testing of new hypotheses about roles of specific genes in particular traits. Existing data from QTL mapping, expression profiling, and early association genetics studies are likely to figure prominently in this merger. To fully realize the fruits of the sorghum sequence, additional functional genomics resources will be needed that provide for identification and study of crippling mutations in specific sorghum genes, in a manner that can be targeted to the subset of genes for which sorghum is a preferred system over rice, maize, or other cereal models.

### 3.1. QTL mapping

Motivated by interest in a range of basic and applied questions, the linkage maps of sorghum have been employed in the “tagging” (mapping) of genes for a large number of traits. The interspecific population has been especially useful for characterization of genes related to domestication, such as seed size, shattering [23], tillering, and rhizomatousness [4]. Plant height and flowering time [35, 36] have been a high priority. Similarly, the importance of hybrid sorghum motivated much research into the genetic control of fertility restoration [37–39]. Resistance genes have been tagged for numerous diseases [40–47], key insect pests [48–51], and also the parasitic weed, striga [40, 52]. Genes and QTLs have been identified that are related to abiotic stresses including postreproductive stage drought tolerance (stay-green) [53–56]; preharvest sprouting [57, 58], and aluminum tolerance [59]. Additional morphological characteristics have also been mapped in interspecific and/or intraspecific populations [21].

### 3.2. Expression profiling

Progress in characterization of the transcriptome has been paralleled by identification of differential gene expression in response to biotic and abiotic factors, including greenbug feeding [60], dehydration, high salinity and ABA [61], and methyl jasmonate, salicylic acid, and aminocyclopropane carboxylic acid treatments [62].

### 3.3. Association genetics

Much of the value of the sorghum sequence may be realized through better understanding of the levels and patterns of diversity in extant germ plasm, which can contribute both to functional analysis of specific sorghum genes and to deterministic improvement of sorghum for specific needs and environments. Sorghum is well suited to association mapping methods because of its medium-range patterns of linkage disequilibrium [63] and its self-pollinating mating system. Extensive *ex situ* sorghum germplasm collections exist within the U.S. National Plant Germplasm System and ICRISAT. Early characterization of complementary association genetics panels developed by a group of US scientists [6], and by Subprogram 1 of the Generation Challenge Program, is in progress. At present, more than 750 SSR alleles and 1402 SNP alleles discovered in 3.3 Mb of sequence [63–66] are freely available from the *Comparative Grass Genomics Center* relational database [67]. Extensive studies of sequence variation in sorghum show that haplotype diversity is low, even when nucleotide diversity is high: for regions of average length 671 bp surveyed in 17 accessions, the median number of haplotypes was three and the mode was two [63]. Common sequence variation can therefore be captured in a small sample of accessions.

### 3.4. Need for mutants and their characterization

A collection of ~400 *S. bicolor* mutants, now under the curation of C. Franks (USDA-ARS, Lubbock TX), provides a start toward testing hypotheses about the functions of individual genes, but a much broader set is needed, ideally providing for the identification of multiple loss-of-function mutants in each gene. Sorghum offers an opportunity to complement more extensive reverse genetics resources in for *Oryza* and *Zea*, providing for the study of genes/gene families that are less tractable in maize or rice (e.g., which remain duplicated in both taxa, but are single copy in sorghum), and also for targeting functional analyses to specific sorghum genes implicated in key traits by association genetics or other approaches.

To accelerate identification in a targeted manner of mutants useful to relate *Sorghum* genes to their functions, 1600 M3 annotated individually pedigreed mutagenized lines using ethyl methane sulfonate have been generated for sorghum genotype BTx623 and their preliminary characterization is in progress [68]. To date, every M3 row inspected closely has been distinguishable from the original stock, and many have multiple mutant phenotypes (Z. Xin, personal communication). More effort in this area is desirable.

Transposon tagging warrants further exploration as a means to obtain additional mutants in sorghum. *Cs1* is the first active transposable element isolated from sorghum, and offers several advantages as an insertion mutagen. *Cs1*-homologous sequences are present in low copy number in sorghum and other grasses, including sudangrass, maize, rice, teosinte, and sugarcane [69]. The low copy number and high transposition frequency of *Cs1* implies that this transposon could prove to be an efficient gene isolation tool. Preliminary studies of *Cs1* as a mutagen (S. Chopra, personal communication) indicate the feasibility of using this transposon as a tagging tool.

## 4. BEYOND SORGHUM-BROADER CHARACTERIZATION OF THE SACCHARINEAE

*Sorghum* sprung from the loins of the Saccharinae group of cereals, which also includes cultivated sugarcane and weedy/invasive Johnsongrass and *Microstegium*. This curious group shows a 6-fold variation in genome size among closely related species with the same chromosome number (*S, bicolor* and *propinquum* versus *nitidum*) [70]; an apparent reduction in chromosome number from the ancestral 20 to 10 in most parasorghums [71]; at least two chromosome doublings in *Saccharum* since its divergence from the remainder of the group [12]; and both natural (*Sorghum halepense*: [4]) and human-mediated polyploidization (*Saccharum* cultivars: [12]). Knowledge of the mechanisms, levels, and patterns of evolution of genome size and structure in this curious group will help to reveal the path by which the sorghum genome has arrived at its present state, also laying the foundation for further study of sugarcane and other economically important members of the group.

Of singular importance is the role that sorghum may play in clarifying the fates and consequences of genes duplicated in recent whole-genome duplications in *Saccharum*, and *Zea* (albeit not in the Saccharinae). *Zea* is the less complicated of these opportunities—a genomewide (or largely so) duplication in the *Zea* lineage shortly followed the *Sorghum-Zea* divergence [14, 72], making *Sorghum* an excellent outgroup for deducing the ancestral state at duplicated loci with regard to location, sequence, regulatory and other features. This opportunity is less complicated in that *Zea* is relatively advanced in restoration of the diploid state with regard to chromosome pairing, behaving for practical purposes as a diploid. *Saccharum* offers insight into an earlier stage following polyploid formation, behaving largely as an autopolyploid although with varying degrees of preferential pairing in different taxa and crosses [12, 73, 74]. *Sorghum halepense*, although far less well studied than either *Zea* or *Saccharum*, appears to be even closer to polyploid formation, in that its formation postdates the divergence of *S. bicolor* and *S. propinquum* which we roughly estimate to be 1-2 million years ago (based on ~1.2% divergence of coding nucleotides). While it is very possible that these three polyploidizations differed in the degree of pairing specificity that was possible at the outset of polyploid evolution, insight into the relative degrees of duplicate gene loss, and/or silencing would be a valuable resource toward clarifying recent hypotheses about adaptation of genomes to the polyploid state [75].
